# Phonological Awareness Skills in Thai‐Speaking Children: A Scoping Review

**DOI:** 10.1111/1460-6984.70099

**Published:** 2025-07-29

**Authors:** Ketnipa Ratanakul, Joanne Cleland, Wendy Cohen

**Affiliations:** ^1^ Department of Psychological Sciences and Health University of Strathclyde Glasgow UK; ^2^ Faculty of Medicine Ramathibodi Hospital, Department of Communication Sciences and Disorders Mahidol University Bangkok Thailand

**Keywords:** lexical tone awareness, phonological awareness, reading, scoping review, Thai‐speaking children

## Abstract

**Background:**

Phonological awareness is an important skill for literacy development. However, limited research has been conducted on tonal languages or non‐alphabetic orthographies, including Thai. Understanding the development of phonological awareness in Thai‐speaking children is important for identifying risk factors for dyslexia and for understanding the role of phonological awareness in Thai‐speaking children with communication impairments.

**Aims:**

This scoping review synthesised empirical studies on phonological awareness in Thai‐speaking children. We aimed to describe the tasks and experiments typically used in studies, outline the performance of phonological awareness in typically developing and non‐typically developing children across preschool to school age, and highlight the importance of phonological awareness skills to literacy development.

**Methods:**

Peer‐reviewed papers were retrieved from eight electronic databases, supplemented by manual reference and website searches for relevant articles on phonological awareness in Thai‐speaking children. The inclusion criteria for eligible studies were articles published in English since 2000 investigating phonological awareness skills in preschool‐ and school‐aged Thai‐speaking children (2;0–12;0 years).

**Main Contributions:**

Fourteen full‐text articles were screened, and 13 met the inclusion criteria. Papers focused on the relationship between phonological awareness and reading skills (*n* = 5), phonological awareness skills in children at risk of learning disabilities or dyslexia (*n* = 5), and lexical tone awareness (*n* = 2). The findings showed that phonological awareness was important for reading abilities across all reported ages, while lexical tone awareness significantly impacted reading abilities only when children were in kindergarten (3;0–6;0 years). Phonological awareness tests were also used as a tool for identifying children at risk of learning disabilities or dyslexia. Initial phoneme identification was the most commonly used phonological awareness task across all 13 studies.

**Conclusions:**

Phonological awareness is essential for reading skills and identifying children at risk of learning disabilities or dyslexia. Future research is needed to investigate the acquisition of phonological awareness in Thai‐speaking children and to examine the phonological awareness skills of children with communication impairments, such as speech sound disorders.

**WHAT THIS PAPER ADDS:**

*What is already known on this subject*

- Phonological awareness is essential for the development of literacy skills across many languages and orthographies. Phonological awareness acquisition in English and other similar languages follows a predictable pattern of development from larger units, such as syllable identification, to smaller units, such as phoneme identification. Children with speech sound disorders, or those at risk of dyslexia, may have problems with these skills, leading to literacy problems or speech sound disorders that are slow to resolve.

*What this paper adds to the existing knowledge*

- This scoping review revealed that phonological awareness is an important element in developing reading skills across ages in Thai‐speaking children. Moreover, lexical tone awareness, which is important in Thai, influenced reading skills only at the kindergarten level, while the effect dissipated in older age groups. A variety of phonological and tone awareness tasks were applied to identify children at risk of learning disabilities or dyslexia in the studies included in the review.

*What are the practical and clinical implications of this work?*

- The findings of this scoping review underscore the importance of phonological awareness in reading skills for Thai‐speaking children. There is currently no single Thai phonological awareness assessment that covers all aspects of phonological and tone awareness for Thai speakers. Additional research is needed on phonological awareness in children with speech, language, and communication needs, such as children with speech sound disorders or developmental language disorder.

## 1 Introduction

Phonological awareness (PA) is the ability to recognise and manipulate the sounds in spoken words (Gillon 2007; Stahl and Murray 1994). It is often described as an essential pre‐cursor to literacy, since children need to recognise that words are made up of smaller units, which in turn have orthographic representations. PA can be categorised into hierarchical levels starting with syllable awareness, followed by onset‐rime awareness, and then phoneme awareness. At each of these levels linguistic complexity can vary (Gillon 2007). The basic level of PA involves the capability to identify the sound structure, for instance, splitting words into syllables (e.g., *com‐pu‐ter*; *e‐le‐va‐tor*), recognising words that share the same rime (e.g., *ham* and *jam*; *gun* and *sun*), and finding words that have the same onset (e.g., *cake* and *cow*; *boat* and *bee*) (Schuele and Boudreau 2008). The more complex level of PA is manipulating individual speech sounds in words—known as phonemic awareness—for example, blending sounds into words (e.g., /k/ /æ/ /t/ into /kæt/ *cat*), segmenting words into sounds (e.g., /pɪɡ/ *pig* into /p/ /ɪ/ /ɡ/), and deleting initial or final sound of words (e.g., deleting the initial sound, /bɒl/ *ball* into /ɒl/) (Schuele and Boudreau 2008).

The development of PA tends to follow the order of complexity of linguistic structure; that is, word awareness is the first skill children acquire, followed by syllable awareness, onset‐rime awareness, and then phoneme awareness (Anthony et al. 2003; Carroll et al. 2003). Phoneme awareness, the more complex level of PA, probably develops around preschool age, at approximately 4 years and 9 months (Carroll et al. 2003). Anthony et al. (2003) also suggests that when the linguistic complexity is controlled, children can become proficient in blending before segmenting the sound structure of a language. Moreover, children can acquire different PA skills at the same time. For instance, children who are capable of blending onsets and rimes might start to know how to blend phonemes (Anthony et al. 2003). The acquisition of PA is essential for learning to read. (Anthony and Francis 2005). The starting point of reading skills in young children is expected to occur when they acquire basic PA skills—such as recognising the beginning and ending sounds in words and rimes—and letter knowledge (Anthony et al. 2003). Children who have difficulty with these skills will have difficulties learning to read and may be at risk for dyslexia since dyslexia is a difficulty with phonological processing, and one of the essential competencies of phonological processing is PA (Anthony and Francis 2005). Additionally, some children with speech sound disorders struggle with both speech production and PA due to deficits in phonological representation, which impacts their PA performance (Sutherland and Gillon 2007).

Studies on PA have investigated various areas, including the relationship between PA and reading abilities (e.g., Burnham et al. 2011; Stahl and Murray 1994), the assessment of PA skills in children with dyslexia (e.g., Anthony and Francis 2005; Roongpraiwan et al. 2002) or speech sound disorders (e.g., Rvachew 2006), and the development of PA (e.g., Anthony et al. 2003; Carroll et al. 2003). One key finding regarding the relationship between PA and reading is that knowledge of onset and rime tends to be associated with reading abilities, particularly once children acquire letter identification skills (Stahl and Murray 1994; Anthony et al. 2003). In the context of dyslexia, assessing PA is crucial for early detection (Thongseiratch et al. 2023), as children with dyslexia are likely to perform poorer than typically developing (TD) children (Roongpraiwan et al. 2002). Moreover, McBride‐Chang (1995) demonstrated three major components of PA: speech perception, cognitive ability, and verbal short‐term memory, with speech perception being the most critical to PA. Similarly, Rvachew (2006) has suggested that speech perception is one of the most significant predictors influencing PA in children with speech sound disorders.

Studies investigating PA have applied various PA tasks in their experiments. For example, PA tasks in the study by Stahl and Murray (1994) included phoneme blending, segmentation, isolation, and deletion. Each task involved four stages of linguistic complexity. For instance, phoneme blending consisted of four stages: onset‐rime blending, vowel–coda blending, blending cluster onsets, and blending cluster codas. Anthony et al. (2003) employed blending and deletion tasks with four degrees of linguistic complexity—blending or deleting words, syllables, onset‐rime, and phonemes—to evaluate participants' PA abilities. Moreover, matching tasks have been used to assess PA (e.g., Bird et al. 1995; Carroll et al. 2003; Rvachew 2006). For instance, in the study by Carroll et al. (2003), participants completed PA matching tasks where they selected a picture from a two‐forced‐choice setup to match a target picture based on various instructions, including rime, initial syllable, final syllable, and initial phoneme matching. Another method to assess PA is the odd‐one‐out task. For example, in research by Liao et al. (2016), the initial phoneme isolation task required asking participants to identify which one of four words had a different initial consonant from the others. The stimuli used in PA studies include real words (e.g., Carroll et al. 2003; Shu et al. 2008; Stahl and Murray 1994) and pseudowords (e.g., McBride‐Chang 1995). Pseudowords have been used to prompt participants to rely on phonological strategies rather than orthographic strategies (Stuart 1990).

Across languages, the development of PA is influenced by the type of language and type of orthographic system (Anthony and Francis 2005). For instance, in English, which has alphabetic orthography, phoneme awareness significantly influences early literacy acquisition (McNeill and Gillon 2020). Moreover, the types of alphabetic orthography—whether transparent (e.g., German, Dutch) or inconsistent (e.g., English, French)—also play a significant role in PA development. For example, German‐speaking children can develop phoneme awareness earlier than English‐speaking children during their first year of school due to the more transparent orthography (Anthony and Francis 2005). In contrast, in Japanese, a syllabic orthography, children performed better in syllable awareness than phoneme awareness tasks and could segment spoken words using a syllable‐based approach (Defior 2004). Less work has been undertaken on tonal languages, e.g., Chinese and Thai. Lexical tone is a suprasegmental feature that differentiates meanings between words with the same phonological structure (Shu et al. 2008; Winskel and Iemwanthong 2010). Tones in Thai are represented in orthography, while tones in Chinese are not. However, tones are represented in Pinyin, an alphabetic script used to write Chinese, which has tone diacritics (Burnham et al. 2011). Accordingly, one of the essential PA abilities related to word reading for young children who speak a tonal language, where tone is represented in the orthography, is lexical tone awareness (Shu et al. 2008; Thongseiratch et al. 2021). Although knowledge of PA is mostly well‐established in English‐speaking populations, there is a need to focus more on investigating PA skills in children who speak tonal languages, such as Thai.

Thai script is an abugida system, which means consonant symbols represent a consonant plus default vowel, and diacritic marks are used to indicate different vowels and tones (Ding et al. 2018). For instance, in the example by Aroonmanakun (2008), the default vowel in a syllable without a coda is /a/. In the word ขนุน (/kʰànǔn/), meaning *jackfruit*, the consonant letter ‘ข’ (kʰ) includes the default vowel /a/, so it is pronounced /kʰà/. It has a low tone because /kʰ/ is a high‐class consonant in a syllable with a short vowel, no coda, and no tone diacritic, which always means the syllable is read with a low tone. In the following syllable, the vowel diacritic mark ‘ุ’ (/u/) is added below the consonant letter ‘น’ (/n/) to produce the word ‘นุน’ as /nǔn/, and it is a rising tone because it is preceded by the high‐class consonant (kʰ).

Thai has 44 consonant letters (Vibulpatanavong and Evans 2019) representing 21 phonemes (21 initial and 9 final phonemes) (Tingsabadh and Abramson 1993) and 32 vowel letters (Vibulpatanavong and Evans 2019) representing 21 phonemes (Tingsabadh and Abramson 1993). Thai has five tones: mid, low, rising, high, and falling, with four diacritics used to indicate tone (Vibulpatanavong and Evans 2019). A Thai syllable has four main components: onset, nucleus, coda, and tone, in which the nucleus and tones are obligatory (Noobutra 2019), while the initial consonant cluster can have up to two consonants, and the coda can have only one consonant (Vibulpatanavong and Evans 2019).

There have been a limited number of studies on PA in Thai (e.g., Thongseiratch et al. 2021; Yampratoom et al. 2017). Therefore, the objective of the current paper is to review available studies that have developed PA assessments or interventions designed specifically for Thai‐speaking children, given the lack of any systematic review on this topic.

## 2 Method

A scoping review methodology was selected instead of a systematic review because PA in tonal languages is an emerging area, and study methods were likely to be diverse. Scoping review methodology allowed us to gather a broad overview of the area and identify any emerging gaps in the literature. The aims of this scoping review therefore include determining the key concepts related to the topic, discovering the available forms of evidence, and identifying any gaps in the existing literature (Mak and Thomas 2022). The study followed the guidelines of the PRISMA extension for scoping reviews (PRISMA‐ScR) (Tricco et al. 2018).

### 2.1 Research Question

The research question was formulated using the Population, Concept, Context framework recommended by the Joanna Briggs Institute for scoping reviews (Peters et al. 2015). Scoping review methodology allows a broad research question or topic, which is preferred for mapping the literature. Therefore, the review question was: What is the performance of Thai‐speaking children in PA experiments across preschool and school‐age years? Where Population = Thai‐speaking children, Concept = PA experiments, and Context = Thai schools (e.g., nursery, kindergarten, and primary schools).

### 2.2 Identifying Relevant Articles

The original search was performed in February 2024 using eight electronic databases: Web of Science, Medline (ProQuest platform), Medline (Ovid platform), Linguistics and Language Behavior Abstracts (LLBA), APA PsycInfo, CINAHL, Scopus, and Child Development & Adolescent Studies. Re‐run searches were conducted in December 2024 to ensure the inclusion of any newly published articles. Manual reference searching of the included papers and searching on websites (Google and Google Scholar) were also done to ensure as many studies as possible were included. The search terms were based on Boolean operators (see Table 1).

**TABLE 1:** Search terms for each database.

Database	Search terms
APA PsycInfo	(“phonological awareness” OR “phoneme awareness” OR lexical tone awareness OR lexical tone perception) AND (Thai* N3 (child* OR school* OR preschool*))
CINAHL	
Child Development & Adolescent Studies	
Medline (ProQuest platform)	(“phonological awareness” OR “phoneme awareness” OR lexical tone awareness OR lexical tone perception) AND (Thai* NEAR/3 (child* OR school* OR preschool*))
Linguistics and Language Behavior Abstracts (LLBA)	
Web of Science	
Medline (Ovid platform)	(“phonological awareness” OR “phoneme awareness” OR lexical tone awareness OR lexical tone perception) AND (Thai* ADJ3 (child* OR school* OR preschool*))
SCOPUS	(“phonological awareness” OR “phoneme awareness” OR lexical tone awareness OR lexical tone perception) AND (Thai* W/3 (child* OR school* OR preschool*))

The inclusion criteria for selecting eligible studies were deliberately broad due to the potential limited number of studies of PA in Thai‐speaking children. However, the specific criteria were:

1. Studies involving both TD children and those who have atypical development (e.g., children with speech sound disorders)
2. Peer‐reviewed academic journal articles, conference papers, and theses
3. Published in English (The search was restricted to papers published in English because the second reviewer is not a Thai speaker.)
4. Published after 2000
5. Participants' ranged from preschoolers to school‐aged children. Due to the varying definitions of ‘preschooler’ and ‘school‐aged,’ the age range was defined according to Kail (2011), who categorises preschoolers as ages 2;0 to 5;11 years and school‐aged children as ages 6;0 to 12;0 years. Studies with participants aged 2;0 to 12;0 were therefore included.
6. Experiments conducted in any location

The exclusion criteria were studies that were not published in English, did not examine PA, or did not involve Thai‐speaking children.

**FIGURE 1:**
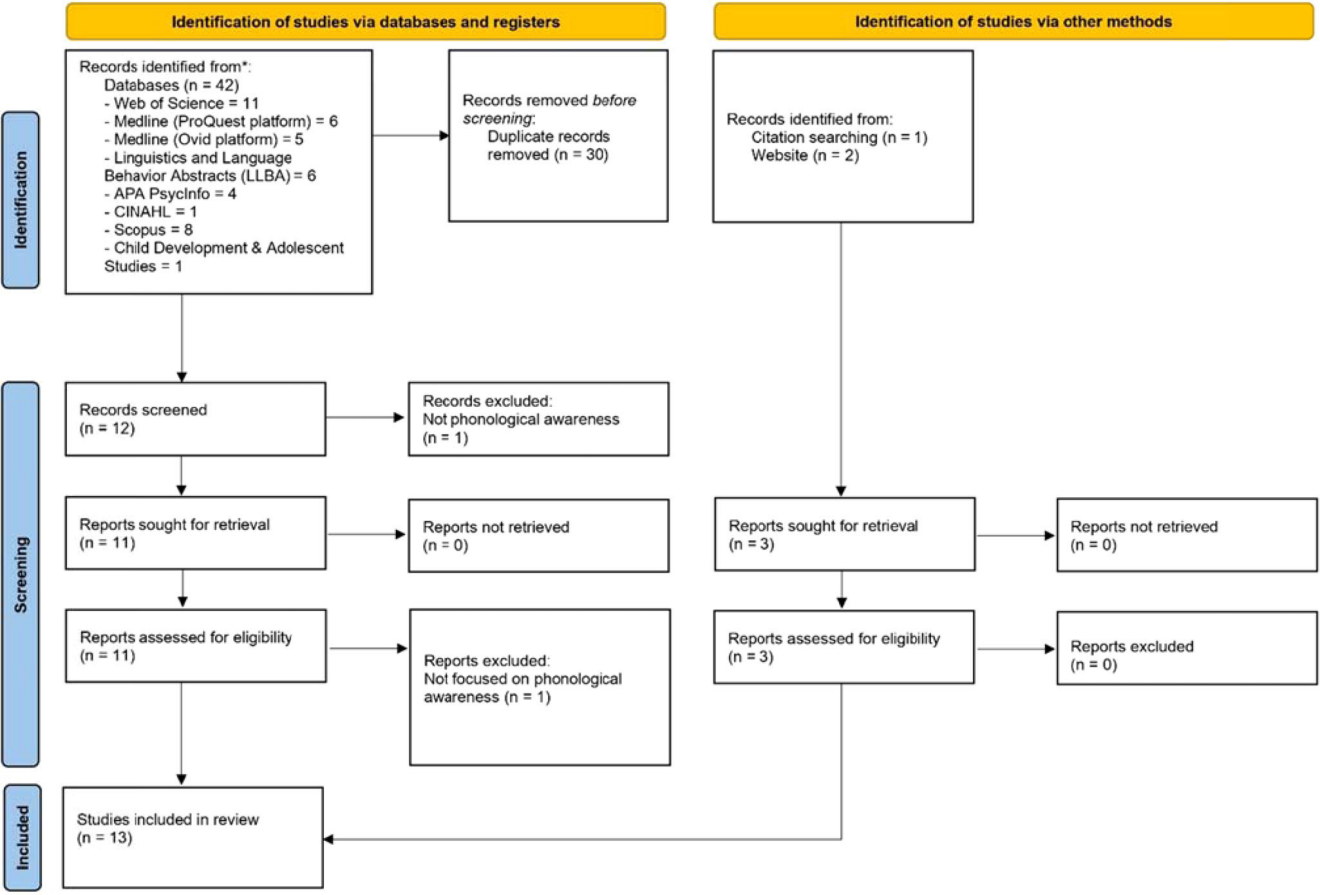
A PRISMA flowchart illustrating the search procedure.

An initial search yielded 42 papers from electronic databases, one from a citation search, and two from websites. The screening process and inclusion decisions were conducted on Rayyan, a web‐based tool (Ouzzani et al. 2016) that supports the screening process of scoping reviews. After removing 30 duplicates, two reviewers (the first and second authors) screened the title and abstract of the remaining 15 papers, and then 14 full‐text papers were retrieved and screened based on inclusion and exclusion criteria. Ultimately, 13 papers were included, and inter‐rater reliability was assessed during screening and data extraction, with 100% agreement between the reviewers. One paper was excluded during the title and abstract screening because it was not relevant to PA, while another was excluded during the full‐text review as it focused on writing rather than PA. The PRISMA flowchart in Figure 1 details the selection process.

### 2.3 Data Extraction

Data were extracted into three custom Microsoft Excel spreadsheets. The first table contained the extracted data from the final 13 articles included in this scoping literature review. The details extracted were author(s), publishing year, study objectives, participants, methods, experiment tasks, and main findings (see Table 2). The second table outlines the definitions and examples of each PA task (see Table 3), and the third table summarises the types of PA tasks used in each study (see Table 4).

**TABLE 2:** Summary of included papers.

Authors	Objectives	Participants	Method	Tasks	Main findings
Roongpraiwan et al. (2002)	To investigate the prevalence of dyslexia in Thai primary‐aged children and describe their clinical characteristics. Characteristics examined were neurological signs, gender, verbal intelligence, and comorbidity with attention deficit hyperactivity disorder (ADHD). This study also aimed to create a Thai screening test for reading and PA skills.	486 children in Grades 1 to 6 were recruited from a school in Bangkok. Precise age range was not specified.	All children were evaluated for nonverbal intelligence using Raven's Standard Progressive Matrices. Children who scored below the fifth percentile were excluded. Each child was assessed for their reading and PA skills using a screening test developed in the study. In addition to reading and PA skills, each child was examined for neurological status, verbal intelligence, and comorbidity with ADHD.	There were five tasks in the screening test. ‐ Reading unfamiliar words ‐ Reading single words ‐ PA tasks: Each child was asked to say the remaining part of a word after omitting a particular phoneme. ‐ Writing spoken word ‐ Reading comprehension There were three assessments for the clinical characteristics. ‐ A paediatric neurologist examined the neurological status of each child. ‐ The verbal part of WISC was used to assess verbal intelligence. ‐ DSM‐IV criteria, Conners parents rating scales, and Conners teachers rating scales were used to assess comorbidity in ADHD.	1. The prevalence of dyslexia was 6.3%, and the gender ratio was 3.4:1 (male: female). 2. The mean Thai reading and PA scores in the dyslexia group were significantly lower than those in the TD children group. 3. The gross neurological examinations of the dyslexia group were normal. However, 90% of them exhibited positive soft neurological signs. 4. The dyslexia group had a lower mean verbal IQ on the WISC test than the TD children group. 5. 8.7% of the dyslexia group had comorbid ADHD.
Wei (2005)	This dissertation investigated the relationship between PA and reading abilities in Thai primary school children using Thai and English assessments. The transfer from Thai PA to English PA skills was also observed.	424 Thai students in Grade 3 were recruited from schools in lower northern Thailand. Precise age range was not specified.	English assessments were conducted first, followed by Thai assessments.	Focusing on Thai assessments Thai PA tasks: ‐ Initial phoneme identification ‐ Final phoneme identification ‐ Rime awareness ‐ Phoneme deletion Thai reading abilities tasks: ‐ Letter identification ‐ Real word reading ‐ Pseudoword reading.	1. Predictors for Thai real‐word reading were final phoneme identification, phoneme deletion, and initial phoneme identification. Final phoneme identification was the strongest predictor. 2. Predictors for Thai pseudoword reading were phoneme deletion, final phoneme identification, and initial phoneme identification. Phoneme deletion was the strongest predictor.
Burnham et al. (2011)	To investigate the perceptual and psycholinguistic representation of PA and tonological awareness (TA). There were three experiments: 1. Thai participants 2. Cantonese participants 3. English‐language participants. Thai stimuli were used in all three experiments to examine the difference between tonal language (Thai and Cantonese) and nontonal language (English). Thai differs from Cantonese, as Thai has a specific orthography for phones and tones. The association of reading skills and PA and TA was also investigated.	Experiment 1: PA and TA in Thai participants, 48 children aged 4;10‐12;2 were divided into four groups: kindergarten, Grade 2, Grade 4, Grade 6. 24 adults were divided into two groups: completed primary education, and completed tertiary education.	Each participant received odd‐one‐out tasks: one phone and tone block, three phone blocks, and three tone blocks. A Thai reading test was used to evaluate their reading skill.	Odd‐one‐out tasks were used to test PA and TA. There were three Thai words in each item, and the participants were asked to choose one that was different. —One phone and tone block The tone and/or medial vowel were the key differences. ‐ Three phone blocks The medial vowel was the key difference. ‐ Three tones blocks The tone was the key difference. Thai reading test ‐ 40 short sentences.	1. Thai participants performed better in PA than TA tasks across ages, which can imply that phones and tones are perceived differently. 2. PA was better than TA, maybe because of the different orthographic systems for phones and tones in Thai. 3. PA and TA positively correlated with reading abilities in Thai children regardless of age.
Onsuwan et al. (2014)	To investigate the performance of Thai children in lexical tone perception tasks.	75 children were divided into three groups by age: 2;0‐3;11, 4;0‐5;11 and 6;0‐7;11 years. Five adults aged 20–25 were recruited as a control group.	There were 10 target tone pairs and five filler word pairs. The participants were asked to choose a target word after listening to a speech stimulus via headphones.	Perception of lexical tones task ‐ Each item was a two‐picture forced choice. ‐ These two choices were minimal pairs (only lexical tone difference).	1. The oldest child group achieved the best scores, followed by the middle and then the youngest groups. The age seemed to matter. 2. A low tone was the most confusing, as the children responded to it as a mid‐tone.
Liao et al. (2016)	To examine cognitive components involved in reading in Thai.	1181 students in Grade 4, aged between 9;2 and 11;9 years, were recruited from schools in one of the provinces in northeastern Thailand.	There were six assessments for evaluating the cognitive components of reading.	‐ PA task: odd‐one‐out (identifying the word with a different initial phoneme) ‐ Rapid word segmentation ‐ Rapid colour naming ‐ Rapid number naming ‐ Morphological structure ‐ Morphological production.	1. Morphological awareness, PA, and rapid naming skills are important cognitive components for reading in Thai.
Yampratoom et al. (2017)	To investigate pre‐reading skills in Thai preschool children and explore the essential factors related to pre‐reading skills.	412 children in kindergarten Year 3, aged 60 to 71 months, were recruited from schools in an urban city in Thailand.	There were experiments and questionnaires. The questionnaires collected from parents and teachers covered important factors related to pre‐reading skills, e.g., socioeconomic data, mother's education, and reading activities per week.	‐ PA task: Initial phoneme identification (Rama Pre‐Read (RPR) program) ‐ Letter naming: naming letters and naming letters with pictures.	1. Participants got an average score of 4.5 out of 10 on the initial phoneme identification task. 2. The average score for naming letters with pictures was higher than for letters without pictures. 3. Maternal education and family income were essential to initial phoneme identification and letter‐naming skills. However, gender and reading activities at home were associated with only letter‐naming skills.
Munthuli et al. (2019)	To develop a paper‐based screening test for learning disabilities (LD) designed for Thai children at risk of LD.	Two groups of participants from Grades 1–4 were recruited from schools in Bangkok, Thailand: 10 TD children and 5 children with LD. Precise age range was not specified.	Before the experiment session, the participants were tested using standard tests, such as WISC‐III, KUS‐SI, or Test of Nonverbal Intelligence, Fourth Edition (TONI‐4).	PA tasks: ‐ Initial phoneme deletion ‐ Phoneme discrimination ‐ Phoneme identification ‐ Phoneme substitution ‐ Rime awareness.	1. Children with LD scored around 50% below TD children on PA tasks. 2. Initial phoneme deletion and phoneme substitution tasks were too difficult for both groups. 3. PA is a good measure for screening LD.
Vibulpatanavong and Evans (2019)	To investigate the association between reading and PA skills as well as the development of PA and reading skills in Thai students.	310 children were recruited from schools in Bangkok and were divided into three groups: Grades 1, 2, and 3. Precise age range was not specified.	There were two experiment sessions: 1. PA tasks, and 2. letter knowledge measure and reading ability tasks. The questionnaire about children's backgrounds was collected from parents.	‐ PA tasks: initial phoneme identification, rime awareness, non‐word blending, non‐word segmenting, final phoneme deletion ‐ Letter knowledge ‐ Word reading ‐ Non‐word reading ‐ Passage reading (reading comprehension).	1. Compared to the final phoneme deletion task, Thai children performed better on the initial phoneme identification and rime awareness tasks. 2. Across three groups, initial phoneme identification was a consistent predictor of reading skills (word reading and letter knowledge). 3. Rime awareness was the best predictor for reading abilities (word, non‐word, and passage reading) in Grade 3 but not in Grade 1 and Grade 2.
Munthuli et al. (2020)	To develop a computer‐based screening test for LD designed for Thai children at risk of LD.	Two groups of participants were recruited: 60 TD children aged 6–8 years from a school in Bangkok and 50 children with LD aged 7–11 years from a hospital in Bangkok.	Before the experiment session, TD children had their IQ assessed using the Test of Nonverbal Intelligence, Fourth Edition (TONI‐4), while the IQ of children with LD was evaluated using the Wechsler Intelligence Scale for Children‐Fourth Edition (WISC‐IV) and WRAT‐Thai. The Strengths and Difficulties Questionnaire (SDQ) for TD children was collected from parents and teachers.	‐ Rapid naming (RN) ‐ Decoding (DEC) ‐ Morphological awareness (MA) ‐ PA tasks: initial phoneme deletion, phoneme discrimination, phoneme identification, phoneme substitution, rime awareness ‐ Mathematics (MAT) ‐ Memory (MEM).	1. This computerised test could classify children with LD and TD. 2. There was a significant difference between TD children and children with LD in PA, MA, DEC, and RA, with PA showing the most significance. 3. For PA subtests, only the phoneme discrimination did not show a significant difference.
Thongseiratch et al. (2021)	This longitudinal study investigated the effect of lexical tone awareness (LTA) on literacy skills and early word recognition at the kindergarten level and the relationship between LTA and word reading and spelling skills at Grade 3 level.	330 children were recruited from schools in southern Thailand. The study had two phases: one during kindergarten and the other in Grade 3. Precise age range was not specified.	In the first phase, children were assessed on non‐verbal IQ, vocabulary, letter knowledge, rapid automatised naming, PA, LTA, and word recognition. In the second phase, their word reading and single‐word spelling dictation skills were evaluated.	‐ Non‐verbal IQ ‐ Vocabulary ‐ Letter knowledge ‐ Rapid automatised naming (RAN) ‐ PA task: Initial phoneme identification (RPR program) ‐ LTA tasks: lexical tone identification task, lexical tone differentiation ‐ Word recognition ‐ Word reading ‐ Single‐word spelling dictation.	1. Lexical tone identification, letter knowledge, PA, and RAN were predictors for word recognition at the kindergarten level. 2. LTA was not a predictor for word reading or single‐word spelling dictation in Grade 3. However, letter knowledge, RAN, and PA were found to be predictors.
Chowsomchat et al. (2023)	To investigate the association between touchscreen devices and pre‐literacy skills in Thai preschoolers.	317 children aged 5 to 6 years were recruited from primary schools in southern Thailand. They were divided into two groups: 252 children using touchscreen devices (tablets, mobile phones, or both), and 65 children not using any touchscreen devices.	Before the assessment, a questionnaire was used to collect demographic information about the family and details about the child's use of touchscreen devices, including the type and duration.	‐ PA task: Initial phoneme identification (RPR program) ‐ Rapid automatised naming ‐ Letter naming.	1. In every emergent literacy ability, the children who used touchscreen devices performed better than the non‐touchscreen user group. 2. The children using only tablets showed the highest scores in the PA task.
Thongseiratch et al. (2023)	This longitudinal study developed the Rapid Automatised Naming, Phonological Awareness, and Letter Identification (RAPALI) flowchart for identifying children with a risk of dyslexia.	330 children were recruited from schools in southern Thailand. The study had two phases: one during kindergarten and the other in Grade 3. Precise age range was not specified.	Nonverbal IQ, PA, letter identification, and rapid automatised naming tests were used to evaluate participants in the first phase. In the second phase, participants were assessed for their risk of dyslexia using a reading subtest in the Wide Range Achievement Test (WRAT‐Thai).	‐ Nonverbal IQ ‐ PA task: Initial phoneme identification (RPR program) ‐ Letter identification (LI) ‐ Rapid automatised naming (RAN) ‐ Reading test.	RAPALI flowchart Steps in the flowchart for classifying children with a risk of dyslexia 1. RAN—getting high scores (≥ 60 letters per minute) means no risk, but getting low or standard scores (< 60 letters per minute) moves to step 2. 2. PA—getting high scores (> 6 points) means no risk, but getting low or standard scores (≤ 2 points) moves to step 3. 3. LI—getting high scores (≥ 40 letters) means no risk, but getting low or standard scores (< 40 letters) means there is a risk of dyslexia.
Wannapaschaiyong et al. (2023)	To evaluate the efficiency of an early intervention software, SIPLE, in Thai preschoolers at risk of dyslexia.	73 children in kindergarten aged 60 to 66 months were recruited from schools in Bangkok. After the assessment, 16 children were identified as having a risk of dyslexia.	Before the assessment, parents were asked about their child's demographic details and family background. The participants were evaluated for their language development using the Denver II and Mullen Scales of Early Learning (MSEL). Reading abilities were assessed to identify dyslexia risk in participants with normal language development. Participants at risk of dyslexia received early intervention training and were assessed on their reading abilities 1 week after the end of interventions.	Pre‐ and post‐early literacy assessment: ‐ PA task: Initial phoneme identification (RPR program) ‐ Letter knowledge ‐ Rapid letter automatised naming Computer‐based early intervention training: 11 lessons of PA and letter naming.	1. Participants at risk of dyslexia demonstrated higher post‐test scores across all subtests of early literacy skills.

## 3 Results

### 3.1 Publication

Thirteen articles published between 2002 and 2023 met inclusion criteria. Around one article was published per year, except for 2019, in which two articles were published, and in 2023, in which three articles were published. The low number of papers suggests that this is an under‐researched area.

### 3.2 Terminology

The term ‘phonological awareness’ was used in 12/13 studies. One study (Onsuwan et al. 2014) investigated only lexical tone awareness. Lexical tone awareness was described using several different terms: tonological awareness (*n* = 1; Burnham et al. 2011), perception of lexical tones (*n* = 1; Onsuwan et al. 2014), and lexical tone awareness (*n* = 1; Thongseiratch et al. 2021).

### 3.3 Study Characteristics

In all but one study, the design was an assessment design (*n* = 12), the remaining study used a pre‐ and post‐intervention design (*n* = 1; Wannapaschaiyong et al. 2023).

Studies covered three main objectives. The first objective was to determine the relationship between PA and reading skills (*n* = 5; Wei 2005; Burnham et al. 2011; Liao et al. 2016; Vibulpatanavong and Evans 2019; Thongseiratch et al. 2021), while two of the studies also included LTA in their assessments (Burnham et al. 2011; Thongseiratch et al. 2021). The second was to describe PA skills in children at risk of LD or dyslexia (*n* = 5; Roongpraiwan et al. 2002; Munthuli et al. 2020, 2019; Thongseiratch et al. 2023; Wannapaschaiyong et al. 2023). The third was to investigate PA (*n* = 3; Yampratoom et al. 2017; Vibulpatanavong and Evans 2019; Chowsomchat et al. 2023), PA and LTA (*n* = 2; Burnham et al. 2011; Thongseiratch et al. 2021), and only LTA skills (*n* = 1; Onsuwan et al. 2014) in TD children. No studies specifically investigated PA in Thai‐speaking children with speech sound disorders.

### 3.4 Participants

**FIGURE 2:**
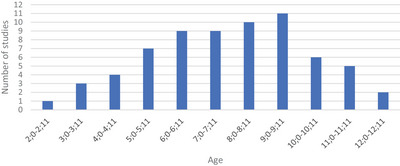
Ages of participants.

Participant ages ranged from 2;0 to 12;0 years, where some studies covered multiple age groups. One study (Burnham et al. 2011) included participants aged up to 12;2 years. Despite this slight extension beyond the inclusion criteria of 12;0 years, the study was included because it contained multiple participant groups that met the inclusion criteria. Six studies did not specify the age of the participants; however, they included the educational stage of the participants, from which we were able to derive approximate ages. Participants aged 9;0–9;11 were the most common group across 13 studies (see Figure 2).

### 3.5 Comparison Groups

Three studies examined performance across age groups (Burnham et al. 2011; Onsuwan et al. 2014; Vibulpatanavong and Evans 2019). For the investigation of PA skills in children with a risk of LD or dyslexia, four studies compared performance between children with LD or dyslexia and a TD children control group (Munthuli et al. 2020, 2019; Roongpraiwan et al. 2002; Wannapaschaiyong et al. 2023). Two studies were longitudinal in nature, with the participants in kindergarten (average age of 67 months) in the first phase and in Grade 3 (average age of 102 months) in the second phase (Thongseiratch et al. 2021, 2023). One study focused on the impact of touchscreen devices on PA skills by comparing children using touchscreen devices with children not using any touchscreen devices (Chowsomchat et al. 2023). The other three studies had only one TD children group as part of their investigation of the relationship between PA and reading skills (Liao et al. 2016; Wei 2005; Yampratoom et al. 2017).

### 3.6 Settings of the Study

All studies conducted the experiments in schools, mainly in Bangkok (*n* = 5; Roongpraiwan et al. 2002; Munthuli et al. 2020, 2019; Vibulpatanavong and Evans 2019; Wannapaschaiyong et al. 2023) and southern Thailand (*n* = 3; Thongseiratch et al. 2021, 2023; Chowsomchat et al. 2023). The other study settings were in northern (*n* = 1; Wei 2005) and northeastern (*n* = 1; Liao et al. 2016) Thailand. One study (Yampratoom et al. 2017) stated that the setting was the schools in an urban city but did not mention specific provinces. The other two (Burnham et al. 2011; Onsuwan et al. 2014) did not specify the setting of the study. It is possible that there are educational performance gaps between urban and rural schools, such as academic resources, financial support, and curricula (Lounkaew 2013); therefore, knowledge about the setting of the study is important.

### 3.7 Types of PA Tasks

Phoneme identification tasks were the most frequently used task in studies (*n* = 9). These comprised the initial phoneme identification task (*n* = 6; Yampratoom et al. 2017; Vibulpatanavong and Evans 2019; Thongseiratch et al. 2021, 2023; Chowsomchat et al. 2023; Wannapaschaiyong et al. 2023) and both initial and final phoneme identification tasks (*n* = 3; Wei 2005; Munthuli et al. 2020, 2019). Regarding the initial phoneme identification task, Yampratoom et al. (2017) created an early reading skills test for assessing emergent literacy skills called Rama Pre‐Read (RPR), which has the initial phoneme identification task as a tool for evaluating PA skills. Five studies (Yampratoom et al. 2017; Thongseiratch et al. 2021, 2023; Chowsomchat et al. 2023; Wannapaschaiyong et al. 2023) used the initial phoneme identification task on the RPR test without any adaptations. This task consisted of one picture as a stimulus, one target picture, and two distractor pictures. The tester named the stimulus word, and then the child was asked to choose the picture of the word with the same initial phoneme as the stimulus. This method of phoneme identification was also found in other studies (e.g., Munthuli et al. 2019; Vibulpatanavong and Evans 2019); however, in these other studies the phoneme identification tasks comprised a different number of distractor words. For example, Munthuli et al. (2020, 2019) designed a PA assessment in which the initial and final phoneme identification tasks consisted of one stimulus picture, one target picture, and one distractor picture. Another study by Vibulpatanavong and Evans (2019) used four picture stimuli: a target picture and three distractor pictures. There was no picture for the stimulus; the tester only said the stimulus word, and the authors did not specify the number of repetitions of the stimulus. Wei (2005) used a different method entirely for their phoneme identification tasks: the tester said the word and then asked the child to name the initial or final phoneme of the word.

Another PA task commonly used was rime awareness (*n* = 4; Wei 2005; Munthuli et al. 2020, 2019; Vibulpatanavong and Evans 2019). Three of the studies (Munthuli et al. 2020, 2019; Vibulpatanavong and Evans 2019) used a similar method: each child was asked to find a picture of a word with the same rime as a stimulus word spoken by the tester. For picture stimuli, in the studies by Munthuli et al. (2020, 2019), there were two picture‐forced choices, and in the study by Vibulpatanavong and Evans (2019), there was one target picture and three distractor pictures. In contrast to the rime awareness tasks mentioned earlier, each participant in the study by Wei (2005) was asked to decide whether two words spoken by the tester shared the same rime.

Regarding the LTA task, there were three methods used. In two studies (Onsuwan et al. 2014; Thongseiratch et al. 2021), lexical tone identification was tested with minimal tone pairs, and the participant was asked to identify a picture to match the word spoken by the tester (Thongseiratch et al. 2021) or heard from an audio recording through headphones (Onsuwan et al. 2014). One study (Thongseiratch et al. 2021) used lexical tone differentiation, with a set of three monosyllabic words that had the same onset and rime but different tones, and each participant was asked to differentiate between tones. The last method used was odd‐one‐out (*n* = 1; Burnham et al. 2011). In this task there were three words, and the participant was asked to judge which word had a different tone.

Phoneme deletion tasks were also used in several studies (*n* = 5; Roongpraiwan et al. 2002; Wei 2005; Munthuli et al. 2020, 2019; Vibulpatanavong and Evans 2019), including initial phoneme deletion (*n* = 3; Wei 2005; Munthuli et al. 2020, 2019), final phoneme deletion (*n* = 2; Wei 2005; Vibulpatanavong and Evans 2019), and one study that did not specify the position of the phoneme deletion (*n* = 1; Roongpraiwan et al. 2002). Each participant was asked to delete the initial or final phoneme of a word and then pronounce the remaining part of the word.

In addition to the PA tasks mentioned above, various other tasks were used. For instance, phoneme discrimination (*n* = 2; Munthuli et al. 2020, 2019), phoneme substitution (*n* = 2; Munthuli et al. 2020, 2019), non‐word blending (*n* = 1; Vibulpatanavong and Evans 2019), non‐word segmenting (*n* = 1; Vibulpatanavong and Evans 2019), and odd‐one‐out (initial or vowel differences) (*n* = 2; Burnham et al. 2011; Liao et al. 2016).

A summary of the definitions and examples of PA tasks in Thai is shown in Table 3. Each example is classified by task type (input or output) and by whether it requires lexical representation access, based on the psycholinguistic framework of speech processing by Stackhouse and Wells (1997). Output tasks require spoken responses; however, if the response is limited to ‘yes’ or ‘no,’ it can be considered a silent response. Input tasks require silent responses, such as pointing, nodding, or shaking the head. A summary of PA tasks used across 13 studies is presented in Table 4.

**TABLE 3:** Definitions and examples of PA tasks in Thai.

PA task	Definition	Example	Input/Output	Lexical representation access
Phoneme identification	This task focuses on identifying a phoneme in either the initial or final position of a word (e.g., Munthuli et al. 2019).	Select the picture with the same initial phoneme as the stimulus. For example, the stimulus is /pʰát/ *fan*. The choices are /pʰɛ́ʔ/ *goat*, /tàw/ *turtle*, and /wɛ̂ːn/ *eyeglasses*. The correct response is /pʰɛ́ʔ/.	Input	Yes
		The word /bâːn/ *house* is presented aloud. The child is asked to say the initial phoneme of the word, which is /b/.	Output	Optional
Phoneme deletion	This task assesses the ability to manipulate phonemes in a word by removing a phoneme and producing a new non/word (e.g., Munthuli et al. 2019).	The word /sôm/ *orange* is presented aloud. The child is asked to delete the initial phoneme, resulting in [ôm] (nonword).	Output	Optional
Phoneme discrimination	This task assesses the ability to discriminate between different phonemes (e.g., Munthuli et al. 2019).	The stimulus is the word /mǔː/ *pig*. The pictures of the minimal pair, /mǔː/ *pig* and /nǔː/ *rat* (differing in place of articulation), are presented, and the child is asked to select the picture that matches the stimulus.	Input	Yes
Phoneme substitution	This task measures the ability to manipulate phonemes in the word by substituting one sound with another to create a new non/word (e.g., Munthuli et al. 2019).	Two words are presented aloud: /mɯ̄ː/ *hand* and /dīː/ *good*. The child is asked to substitute the initial sound of /dīː/ with /m/ from /mɯ̄ː/, resulting in a new word /mīː/ *to have*.	Output	Optional
Rime awareness	This task involves identifying the word that shares the same rime as the target word (e.g., Vibulpatanavong and Evans 2019).	Select the picture with the same rime as the stimulus. For example, the stimulus is /lāː/ *donkey*. The choices are /jāː/ *pill*, /kʰɔ̄ː/ *neck*, and /hǔː/ *ear*. The correct answer is /jāː/.	Input	Yes
		Two spoken words, /rót/ *car* and /mót/ *ant*, are presented, and the child is asked to judge whether the two words share the same rime. The correct answer is 'yes.'	Input	Optional
Lexical tone awareness	Lexical tone identification This task focuses on identifying the word that matches the lexical tone of the stimulus word (e.g., Thongseiratch et al. 2021).	The stimulus is the word /pūː/ *crab*. The pictures of the minimal pair, /pūː/ *crab* and /pùː/ *grandfather* (differing in lexical tone), are presented, and the child is asked to select the picture that matches the stimulus.	Input	Yes
	Lexical tone differentiation This task assesses the ability to distinguish between syllables that share the same onset and rime but differ in lexical tone (e.g., Thongseiratch et al. 2021).	Select the word that has a different tone from the others. For example, a set of monosyllabic words—/nāː/ *field*, /nāː/ *field*, and /nâː/ *face*—is presented aloud, with each word represented by a cartoon face. The correct response is the cartoon face corresponding to the word /nâː/.	Input	Optional
Non‐word blending	This task involves blending sounds to produce a monosyllabic non‐word (e.g., Vibulpatanavong and Evans 2019).	The sounds /tɔ̄ː/ and /ɯː/ are presented aloud. The child is asked to blend the sounds to form the non‐word /tɯ̄ː/.	Output	No
Non‐word segmenting	This task focuses on segmenting a monosyllabic non‐word into separate phonemes (e.g., Vibulpatanavong and Evans 2019).	The non‐word /tɯ̄ː/ is presented aloud and the child is asked to segment it into /tɔ̄ː/ and /ɯː/.	Output	No
Odd one out	This task requires identifying the word in each set that differs from the others in either the initial consonant, vowel, or lexical tone (e.g., Burnham et al. 2011).	Select the odd one out based on the different initial phoneme. For example, the child hears three spoken words: 1. /kʰɔ́ːn/ *hammer*, 2. /kʰàj/ *egg*, and 3. /mɔ̌ː/ *doctor*. The odd one out is 3. /mɔ̌ː/ because it has a different initial phoneme. The child responds by marking the number 3 on the answer sheet.	Input	Optional

*Note*: ‘Optional’ indicates that the task may be completed by either accessing or not accessing the lexical representation.

**TABLE 4:** Types of PA tasks used in each study.

	Type of phonological awareness task														
	Phoneme identification		Phoneme deletion					Lexical tone awareness				Odd one out			
Study	Initial phoneme identification	Final phoneme identification	Initial phoneme deletion	Final phoneme deletion	Phoneme discrimination	Phoneme substitution	Rime awareness	lexical tone identification	lexical tone differentiation	Non‐word blending	Non‐word segmenting	Initial consonant difference	Vowel difference	Vowel and tone difference	Tone difference
Yampratoom et al. (2017)	✓														
Thongseiratch et al. (2021)	✓							✓	✓						
Chowsomchat et al. (2023)	✓														
Wannapaschaiyong et al. (2023)	✓														
Thongseiratch et al. (2023)	✓														
Wei (2005)	✓	✓	✓	✓			✓								
Liao et al. (2016)												✓			
Onsuwan et al. (2014)								✓							
Munthuli et al. (2019)	✓	✓	✓		✓	✓	✓								
Munthuli et al. (2020)	✓	✓	✓		✓	✓	✓								
Vibulpatanavong and Evans (2019)	✓			✓			✓			✓	✓				
Burnham et al. (2011)													✓	✓	✓
Roongpraiwan et al. (2002)			✓^1^												

### 3.8 Speech Stimuli

Across the 13 studies, speech stimuli were verbally provided by the tester (*n* = 11), or pre‐recorded speech was delivered through headphones (*n* = 2; Onsuwan et al. 2014; Munthuli et al. 2020). Additionally, two studies (Onsuwan et al. 2014; Yampratoom et al. 2017) mentioned that their speech stimuli were based on the Central Thai dialect, with the other studies not specifying dialect information.

### 3.9 Summary of Main Findings

#### 3.9.1 PA and Reading Skills

Five studies (Wei 2005; Burnham et al. 2011; Liao et al. 2016; Vibulpatanavong and Evans 2019; Thongseiratch et al. 2021) investigated the relationship between PA and reading skills, and all five reported that PA was a good predictor of reading skills. Two of these (Burnham et al. 2011; Thongseiratch et al. 2021) examined LTA and reading skills. One study (Burnham et al. 2011) applied odd‐one‐out tasks of LTA, and the results showed that LTA correlated with reading abilities in Thai children regardless of age. However, the other study (Thongseiratch et al. 2021) suggested that lexical tone identification (one type of LTA) was a significant predictor for word recognition in kindergarten children (mean age 5;7 years), although it was not a significant predictor for word reading by Grade 3 (mean age 8;6 years).

#### 3.9.2 PA Skills in Children at Risk of LD or Dyslexia

Three studies (Roongpraiwan et al. 2002; Munthuli et al. 2020, 2019) revealed that overall, children with LD or dyslexia performed poorer than TD children in PA tasks. Initial phoneme deletion and phoneme substitution tasks appeared to be too difficult for both groups in Grades 1 to 4 (approximately 6;0 to 10;0 years old) (Munthuli et al. 2019), which suggests a potential floor effect. However, this cannot be confirmed because no detailed performance scores were provided. The authors suggested revising the task design, as they proposed that Thai tonal orthography caused confusion in the initial phoneme deletion task. This is because initial consonant letters represent both onset and lexical tone when writing, but diacritics can be used to change the lexical tone without altering the initial consonant. Therefore, participants tended to delete both the initial phoneme and lexical tone in the initial phoneme deletion task rather than preserving the tone. In general, phoneme deletion tasks may not directly affect the tonal quality of a word in relation to PA, and the word formed after deletion can be either a non‐word or a meaningful word.

Furthermore, there was no significant difference between the two groups in the phoneme discrimination task (Munthuli et al. 2020). Four studies (Roongpraiwan et al. 2002; Munthuli et al. 2020, 2019; Thongseiratch et al. 2023) suggested that PA was a good measure for identifying children with LD or dyslexia. The only intervention study in this review (Wannapaschaiyong et al. 2023) showed that children at risk of dyslexia demonstrated higher PA scores after 11 intervention sessions targeting PA and letter naming.

#### 3.9.3 Children's Performance in Different PA Tasks Across Ages

On average, children in kindergarten aged 5;0 to 5;11 years achieved scores less than 50% correct (4.5 out of 10) on the initial phoneme identification task, which used a three‐alternative forced‐choice design (Yampratoom et al. 2017). Furthermore, the performance on the initial phoneme identification task significantly impacted word recognition for children in kindergarten (average age of 5;7 years) (Thongseiratch et al. 2021). Regarding the effect of touchscreen devices on PA skills in children aged 5;0 to 6;0 years, one study (Chowsomchat et al. 2023) reported that the group of children using touchscreen devices performed significantly better than the non‐use group in the initial phoneme identification task. They suggested that the user‐friendly nature of touchscreen devices makes them more suitable for facilitating young children's performance. Children aged between 6;0 and 8;0 years demonstrated more advanced skills in initial phoneme identification and rime awareness tasks compared to their performance in the final sound deletion task (Vibulpatanavong and Evans 2019). Moreover, the initial phoneme identification task was an essential element of reading skills among children in this age group (Vibulpatanavong and Evans 2019).

In children in Grade 3 (aged approximately 8;0 to 9;0 years), the final phoneme identification was the most significant predictor for real word reading, while phoneme deletion was the best predictive factor for non‐word reading (Wei 2005). In children in Grade 4 (aged approximately 9;0 to 11;0 years), initial phoneme isolation played a crucial role in predicting reading development (Liao et al. 2016).

#### 3.9.4 Children's Performance in Different LTA Tasks Across Ages

One study investigated lexical tone identification (Onsuwan et al. 2014). Results showed that performance improved with age, with the youngest group (2;0–3;11 years) performing the worst while the oldest group (6;0–7;11 years) performed the best. In terms of error analysis, a low tone was most often identified incorrectly as a mid‐tone.

Burnham et al. (2011) applied odd‐one‐out tasks of LTA and compared children's PA and LTA performance. The findings illustrated that children had better PA than LTA across ages (4;10–12;2 years). The authors suggested this could be attributed to the distinct orthographic representation of Thai phonemes and tones.

## 4 Discussion

This scoping review summarises the performance of Thai‐speaking children aged 2;0 to 12;0 years in PA experiments. Thirteen papers were included in the review. PA is a strong predictor of reading skills for Thai‐speaking children, and these results are similar to research on PA and reading in other languages such as English (e.g., Stahl and Murray 1994; Anthony et al. 2003). This is despite these languages being from different language families and despite the orthographic systems differing. One commonly used task to assess PA abilities in Thai‐speaking children was initial phoneme identification (e.g., Yampratoom et al. 2017; Thongseiratch et al. 2021; Chowsomchat et al. 2023). Findings showed that performance on this task was a significant predictor of reading skills across different age groups (Vibulpatanavong and Evans 2019; Thongseiratch et al. 2021). The results from a meta‐analysis on PA in Spanish‐speaking children also showed that initial phoneme identification plays an important role in reading skills (Míguez‐Álvarez et al. 2022). On the other hand, in a longitudinal study, the initial phoneme identification task was not strongly correlated with letter knowledge and reading skills in Arabic‐speaking children (Al‐Sulaihim and Marinis 2017). Variations in findings on this task across languages may result from differences in orthographic systems.

Given that Thai is a tonal language, LTA was also included in the PA experiments (Burnham et al. 2011; Thongseiratch et al. 2021). The results revealed that LTA was a significant predictor for word recognition skills, but only in kindergarten‐aged children (Thongseiratch et al. 2021). This finding is consistent with the research on LTA in Chinese‐speaking children (Shu et al. 2008), which found that LTA was a key predictor influencing word recognition for children at the kindergarten level. Compared to PA studies in other languages, most studies in Thai‐speaking children (*n* = 7, 54%) included only one PA task, which is unlikely to cover all aspects of PA. Moreover, the PA tasks used in the studies reported here were not designed to include varying levels of linguistic complexity, unlike the more comprehensive experimental methods used in English PA research (e.g., Stahl and Murray 1994; McBride‐Chang 1995).

Regarding PA development, Onsuwan et al. (2014) reported that LTA tended to improve with age, and that Thai‐speaking children typically mastered tone‐contrasting skills around the ages of 5;0 to 7;0. This highlights the importance of age in LTA development. Their findings also align with research on LTA in Chinese‐speaking children (Shu et al. 2008), where older children (aged 6;0–7;6 years) showed better LTA abilities. However, no specific research has focused on the development of other PA skills in Thai‐speaking children. Vibulpatanavong and Evans (2019) suggested that Thai‐speaking children in Grades 1 to 3 (approximately 6 to 9 years old) performed better in initial phoneme identification and rime awareness than final phoneme deletion. Therefore, it might be assumed that phoneme deletion skills may develop later than initial phoneme identification and rime awareness skills in Thai‐speaking children. This is consistent with the development of PA in other languages, such as English (e.g., Schuele and Boudreau 2008) and German (e.g., Schaefer et al. 2009), where children achieve onset‐rime awareness before phonemic awareness. Furthermore, PA has been an effective tool for screening Thai‐speaking children at risk of dyslexia or LD, as these children frequently performed poorer than TD children in PA tasks (e.g., Roongpraiwan et al. 2002; Munthuli et al. 2019).

In relation to different methods of assessing PA, these may reflect distinct levels of processing. According to the psycholinguistic framework of speech production by Stackhouse and Wells (1997), PA assessments can be categorised into two types: input and output. Phoneme identification tasks, commonly used in most studies (Yampratoom et al. 2017; Vibulpatanavong and Evans 2019; Thongseiratch et al. 2021, 2023; Chowsomchat et al. 2023; Wannapaschaiyong et al. 2023; Munthuli et al. 2020, 2019), as well as rime awareness tasks (Vibulpatanavong and Evans 2019; Munthuli et al. 2020, 2019), were input tasks, where children responded to the stimulus by pointing to the picture. In this case, pictures were employed to reduce memory load. Children needed to access their semantic representation to identify the picture, then retrieve the phonological structure of the spoken word, and finally, determine which picture shared the same initial phoneme or rime as the stimulus (Stackhouse and Wells 1997). Conversely, the phoneme identification tasks in the study by Wei (2005) were output tasks, where children were asked to produce the initial or final phonemes of the stimulus word given by the tester. In this way, children could complete the task without having to access their lexical representation; however, to assist in completing it, they might use top‐down processing, drawing on stored information from their lexical representation (Stackhouse and Wells 1997). This processing approach can also be applied to the rime awareness tasks by Wei (2005), where children were asked whether two spoken words shared the same rime, with the exception that these tasks were input tasks.

### 4.1 Clinical and Educational Applications

To date, there are no standardised PA assessments in Thai that cover all PA and LTA aspects. Therefore, applying frameworks or assessment designs from other languages could be useful for developing more comprehensive PA assessments in Thai. Standardised PA assessments in English—such as the CTOPP2 by Wagner et al. (2013) and the PHAB2 by Gibbs and Bodman (2014)—include a variety of tasks at different linguistic levels, such as word, syllable, onset‐rime, and phoneme levels. In contrast, existing Thai PA assessments mainly focus on onset‐rime and phoneme levels. Consequently, including word‐ and syllable‐level tasks would enhance the comprehensiveness of Thai PA assessments.

The psycholinguistics framework of speech processing of Stackhouse and Wells (1997) was used to develop PA assessment in German (Schaefer et al. 2009), which included both input and output tasks in each subtest. For example, in the rime awareness input task, the child was asked to select one picture (from a set of three) with the same rime as the stimulus picture. For the output task, the child was asked to say as many words as possible that shared the same rime as the stimulus within 15 s.

When using PA assessments, users should consider which tasks are appropriate for the specific age range (Schaefer et al. 2009; Gibbs and Bodman 2014). Moreover, the selection of word stimuli should be based on words that are commonly found in children's schoolbooks for each age group (Schaefer et al. 2009). For young children, picture stimuli are often used to reduce memory load (Schaefer et al. 2009; Gibbs and Bodman 2014). For example, in the initial phoneme identification task, children aged 5–6 years are asked to choose the picture that begins with the same initial sound as the stimulus, which is presented both as a spoken word and a picture (Gibbs and Bodman 2014).

In the context of Thai dialects and PA assessments, future research is needed to investigate whether dialect differences influence children's performance. Compared to Central Thai, other regional dialects such as Northern Thai, Northeastern Thai, and Southern Thai differ slightly in their consonant inventories and have more tonal distinctions—for example, Northern Thai has six tones, and Southern Thai has seven tones (Siriaksornsat 2002). However, children who do not speak Central Thai as their primary dialect are often exposed to it through school and media.

### 4.2 Limitations

Across the 13 studies included in this scoping review, no studies investigated PA in Thai‐speaking children with speech or language disorders other than LD or dyslexia, such as children with speech sound disorders. Moreover, the order of acquisition of PA with specific ages of typical skill mastery still needs to be investigated, as no studies have concluded the specific age of PA development in Thai‐speaking children.

## 5 Conclusion

This scoping review summarised the PA experiments from 13 articles on Thai‐speaking children. The results revealed that PA is an essential element for reading skills across ages, and LTA influenced word reading only at the kindergarten level. The PA assessment was also an effective tool for identifying children at risk of LD or dyslexia. Among the various PA tasks, initial phoneme identification was the most commonly used. However, further research is needed to explore the development of PA in Thai‐speaking children and PA performance in children with other speech or language disorders, such as children with speech sound disorders or developmental language disorder.

## Conflicts of Interest

The authors declare no conflicts of interest.

## Supporting information




Supplementary information: jlcd70099‐sup‐0001‐PRISMAScRFillableChecklisSUPPLEMt.docx

## Data Availability

All data from the analysis and extraction processes are presented in this published article.

## Note

1 They mentioned only phoneme deletion but did not mention the position of phoneme deletion.
